# Health Related Quality of Life and Influencing Factors among Welders

**DOI:** 10.1371/journal.pone.0101982

**Published:** 2014-07-21

**Authors:** Jingxiang Qin, Wuzhong Liu, Jun Zhu, Wei Weng, Jiaming Xu, Zisheng Ai

**Affiliations:** 1 Baoshan Center for Disease Control and Prevention, Shanghai, China; 2 Shanghai No. 6 People's Hospital, Shanghai, China; 3 Department of Preventive Medicine, College of Medicine, University, Shanghai, China; The Ohio State University, United States of America

## Abstract

**Background:**

Occupational exposure to welding fumes is a serious occupational health problem all over the world. Welders are exposed to many occupational hazards; these hazards might cause some occupational diseases. The aim of the study was to assess the health related quality of life (HRQL) of electric welders in Shanghai China and explore influencing factors to HRQL of welders.

**Methods:**

301 male welders (without pneumoconiosis) and 305 non-dust male workers in Shanghai were enrolled in this study. Short Form-36 (SF-36) health survey questionnaires were applied in this cross-sectional study. Socio-demographic, working and health factors were also collected. Multiple stepwise regress analysis was used to identify significant factors related to the eight dimension scores.

**Results:**

Six dimensions including role-physical (RP), bodily pain (BP), general health (GH), validity (VT), social function (SF), and mental health (MH) were significantly worse in welders compared to non-dust workers. Multiple stepwise regress analysis results show that native place, monthly income, quantity of children, drinking, sleep time, welding type, use of personal protective equipment (PPE), great events in life, and some symptoms including dizziness, discomfort of cervical vertebra, low back pain, cough and insomnia may be influencing factors for HRQL of welders. Among these factors, only sleep time and the use of PPE were salutary.

**Conclusions:**

Some dimensions of HRQL of these welders have been affected. Enterprises which employ welders should take measures to protect the health of these people and improve their HRQL.

## Introduction

Welding is a very important process used for joining metals. With the quick development of science and industry, welding is used in more production fields, and the quantity of welders is increasing. Occupational exposure to welding fumes is a serious occupational health problem all over the world [1]. Welders are exposed to many occupational hazards such as dust, heavy metals, fluoride, ozone, nitrogen oxides, carbon monoxide, and noise, ultraviolet rays during welding. These hazards might cause some occupational diseases such as pneumoconiosis, poisoning, electric ophthalmia, hearing impairment, and so on. Welding fumes are a complex mixture of different metals. Most welding fumes contain a small percentage of manganese [2]. There are many studies concerning about the potential neurological effects associated with exposure to manganese in welding fumes [3]–[8], and some studies explored the association between parkinsonism and exposure to welding fume [9], [10]. However, welders' quality of life has not gotten enough attention, and there are very little literatures about this.

Health related quality of life (HRQL) is an individual's satisfaction or happiness with dimensions of life insofar as affected by “health”. HRQL has been introduced to assess people's health status. To date, a number of questionnaires have been developed to evaluate HRQL, and the 36-item Short Form Health Survey (SF-36) is the most common one [11], SF-36 has been applied to many areas and studies, especially in surveys on HRQL of many patients suffering from different diseases. A study which used SF-36 showed that the quality of life of coal dust worker was significantly lower than ordinary people [12]. Another study which used Chinese World Health Organization Quality of Life-brief version (WHOQOL-BREF) questionnaire also showed low quality of life for coal dust workers without pneumoconiosis in mainland China [13]. A cross-sectional study of UK assessed HRQL of professional divers who had worked as a welder (PDW, *n* = 153), professional welders who had not dived (NDW, *n* = 108), and offshore oil field workers who had neither dived nor welded (NDNW, *n* = 252) with Short Form 12 questionnaire (SF12), and the result showed that health-related quality of life, either physical or mental, did not differ between groups [14]. However, as far as we know, no specific study assessing HRQL of welders with SF-36has been done. In this study, we aim at assessing HRQL of welders in Shanghai, China with SF-36, and evaluating influencing factors to welders' HRQL.

## Methods

### Study population

This cross-sectional study included 301 male welders from 7 factories as welder group, and 305 male general employees from 8 factories as control group in Baoshan district of Shanghai city. Eligibility criteria of welder group included the following: (1) at least one year's welding experience; (2) clinically proven absence of pneumoconiosis (diagnostic criteria of pneumoconiosis was GBZ70-2002 [15]); (3) aged 20 to 55 years old, and without any chronic diseases such as hypertension, diabetes etc. Eligibility criteria of control group were: (1) aged 20 to 55 years old, without exposure to any dust and chemicals; (2) without any chronic diseases such as hypertension, diabetes etc.

### Instruments

The instruments applied in this study consisted of two parts: general information and Mandarin version of SF-36. General information was collected on age, height, weight, native place, educational level, personal monthly income, marital status, quantity of children, tobacco use (smoking), alcohol use (drinking), great events in ten years(i.e. divorce, bereft of one's spouse, jobless, be on a tight budget, strained family relationships), sleep time per night, frequency of exercise per week, and some physical symptoms like cough, headache, dizziness, insomnia, discomfort of cervical vertebra, low back pain, etc. In additional, some information about working years, welding type, local ventilation in workplace, and use of personal protective equipment for welder group was also collected.

The Mandarin version of SF-36 was selected for use in this study because it is one of the most commonly used forms internationally, and it has been tested and proved having good reliability and validity in two surveys of Shanghai [11], [16]. It has 36 items of 8 dimensions of health: physical functioning (PF), role-physical (RP), bodily pain (BP), general health (GH), validity (VT), social function (SF), role-emotional (RE), mental health (MH), and one single item dimension on health transition.

### Data collection and statistical analysis

The study was conducted from January 2011 to November 2012, and used a self-finished interview method. Interviewers were trained by experts from the project group. Workers filled in the questionnaires by themselves and submit to interviewers who examined questionnaires for any errors and ensured the quality of the survey. In SF-36 questionnaire, item scores ranged from 1 to 2, 3, 5 or 6 and were recorded so that all items scored in the same direction, with higher values indicating fewer limitation or better health states [17]. The raw score of each of the eight dimensions was derived by summing the item scores, and transformed to a value for the dimension from 0 (worst possible health state measured by the questionnaire) to 100 (best possible health state) by a formula [11]. 301 from 320 welders and 305 from 320 members of control group completed the questionnaire. The total valid response rate was 94.69%. The 34 incomplete questionnaires with more than 50% of items missing were excluded. All valid questionnaires were doubly input into the database using software Epidata 3.0. Both manual checking and computer checking were conducted to find discrepancies.SPSS20.0 was used for analyzing the survey data. Student t test, One-Way ANOVA, nonparametric Wilcoxon test, and multivariate stepwise regression were applied to evaluate the influencing factors on quality of life.

## Results

The mean age of the welder group (n = 301) was 32.90±8.54, and the mean age of the control group (n = 305) was 34.27±10.03. Table 1 shows general information of the welder group and the control group. There were no significant differences on age, marital status, smoking, drinking, sleep time per night between the groups. However, significant differences were observed on native place, education, personal monthly income, quantity of children and exercise frequency per week between the two groups. The percentage of nonlocal in welder group was higher than that of control group (*P*<0.05), and education level of welder group was lower than that of control group (*P*<0.05). The exercise status of welder group was worse than that of control group (*P*<0.05).

**Table 1:** General information of welder group and control group.

General information		Welder group		Control group		Statistics	*P*
		n	%	n	%		
Age	<30	130	43.2	116	38.0	Z = −1.724	0.085
	30∼45	141	46.8	144	47.2		
	≥45	30	10.0	45	14.8		
Native place	local	78	25.9	110	36.1	*X* ^2^ = 7.296	0.007
	nonlocal	223	74.1	195	63.9		
Education	Primary school	14	4.6	6	2.0	*Z* = −8.516	<0.01
	secondary school	214	71.1	120	39.3		
	high school	73	24.3	179	58.7		
Monthly income	1000∼	41	13.7	45	14.8	Z = −2.888	0.004
	2000∼	135	45.0	172	56.4		
	3000∼	67	22.3	54	17.7		
	4000∼	57	19.0	34	11.2		
Marital status	Single	63	21.0	86	28.2	*X* ^2^ = 7.434	0.059
	Married	228	76.0	216	70.8		
	Divorced and others	9	3.0	3	1.0		
Quantity of children	0	84	27.9	119	39.0	*Z* = −3.629	<0.01
	1	158	52.5	153	50.2		
	2	48	16.0	30	9.8		
	≥3	11	3.7	3	1.0		
Smoking	No	188	62.5	206	67.5	*X^2^* = 1.721	0.190
	Yes	113	37.5	99	32.5		
Drinking	No	222	73.8	210	68.8	*X^2^* = 1.778	0.182
	Yes	79	26.2	95	31.2		
Sleep time per night	<7 h	69	22.9	80	26.2	*Z* = −0.869	0.385
	7∼9 h	220	73.1	213	69.8		
	≥9 h	12	4.0	12	3.9		
Exercise frequency per week	0	143	47.5	102	33.4	*Z* = −3.424	0.001
	1∼2	134	44.5	170	55.7		
	≥3	24	8.0	33	10.8		

In the table 2, we can see almost all the physical symptoms incidental rate of welder group was higher than that of control group. However, nonparametric Wilcoxon test results showed that only in cough, dizziness and low back pain, the differences were significant (*P*<0.05).

**Table 2:** Comparison of physical symptoms between the two groups.

Physical symptoms		Welder group		Control group		Statistics	*P*
		n	%	n	%		
Cough	No	142	47.2	180	59.0	*Z* = −3.177	0.001
	Occasionally	138	45.8	116	38.0		
	Often	21	7.0	9	3.0		
Headache	No	185	61.5	207	67.9	*Z* = −1.698	0.089
	Occasionally	103	34.2	89	29.2		
	Often	13	4.3	9	2.9		
Dizziness	No	175	58.1	213	69.8	*Z* = −3.072	0.002
	Occasionally	111	36.9	84	27.5		
	Often	15	5.0	8	2.7		
Somnia	No	172	57.1	191	62.6	*Z* = −1.532	0.126
	Occasionally	112	37.2	104	34.1		
	Often	17	5.7	10	3.3		
Low back pain	No	111	36.9	161	52.8	*Z* = −4.385	<0.01
	Occasionally	133	44.2	115	37.7		
	Often	57	18.9	29	9.5		
Discomfort of cervical vertebra	No	150	49.8	160	52.5	*Z* = −0.885	0.376
	Occasionally	118	39.2	120	39.3		
	Often	33	11.0	25	8.2		

Table 3 showed that all the means of eight HRQL dimensions of welder group were lower than that of control group, but the differences in six dimensions including role-physical (RP), bodily pain (BP), general health (GH), validity (VT), social function (SF), and mental health (MH) were significant (*P*<0.05).Among the 301 welders, 204 (67.8%) were carbon dioxide (CO_2_) shielded arc welders, 41 (13.6%) were manual arc welders, and the rest 56 welders worked at submerged arc welding, argon arc welding or other types of welding. In Baoshan district, there are many metal processing factories, where carbon dioxide shielded arc welding is the most common welding type. Meanwhile, carbon dioxide shielded arc welding processes usually produce much welding fume. Considering these reasons, we divided welder group into CO_2_ arc welder group and non- CO_2_ arc welder group. The SF-36 scores of the two welder groups were compared with control group in Table 4. One-Way ANOVA results show that the differences of 7 dimensions scores except PF among the three groups were significant (*P*<0.05). Multiple comparisons results show that the means of BP, VT and SF of non-CO_2_ arc welder group were significantly lower than that of control group (*P*<0.05). Compared with control group, the means of 7 dimensions except PF of CO_2_ arc welder group were significantly lower (*P*<0.05). At the same time, the scores of RP, GH and RE of CO_2_ arc welder group were significantly lower than non-CO_2_ arc welder group (*P*<0.05).

**Table 3:** Comparison of SF-36 dimension scores between the two groups.

Dimension	Control group (n = 305)	Welder group (n = 301)	*t*	*P*
PF	90.2±14.0	89.6±13.2	−0.568	0.570
RP	87.3±28.2	77.2±35.6	−3.884	<0.01
BP	88.4±15.7	81.3±19.2	−4.977	<0.01
GH	59.9±8.1	58.1±9.1	−2.557	0.011
VT	68.3±16.4	61.0±19.3	−5.003	<0.01
SF	84.7±16.4	79.9±18.3	−3.442	0.001
RE	85.6±30.5	81.9±33.4	−1.395	0.164
MH	69.4±16.4	64.8±17.9	−3.292	0.001

**Table 4:** Comparison of SF-36 dimension scores among the three groups.

Dimension	Control (n = 305)	Non- CO_2_ arc welder group (n = 204)	CO_2_ arc welder group (n = 97)	*F*	*P*
PF	90.2±14.0	90.2±13.2	89.3±13.3	0.318	0.727
RP	87.3±28.2	83.0±31.0	74.4±37.3**Δ	9.992	<0.01
BP	88.4±15.7	83.9±16.7*	80.1±20.2**	13.952	<0.01
GH	59.9±8.1	59.8±9.8	57.3±8.8**Δ	6.104	0.002
VT	68.3±16.4	63.5±19.3*	59.8±19.3**	13.943	<0.01
SF	84.7±16.4	79.8±19.2*	79.9±17.9**	5.934	0.003
RE	85.6±30.5	89.0±27.1	78.6±35.6* ^ΔΔ^	4.503	0.011
MH	69.4±16.4	67.1±18.4	63.7±17.6**	6.760	0.001

*: Compared with Control, *P*<0.05; **: Compared with Control, *P*<0.01;

Δ : CO_2_ arc welder group Compared with non- CO_2_ arc welder group,*P*<0.05; ^ΔΔ^: CO_2_ arc welder group Compared with Non- CO_2_ arc welder group, *P*<0.01.

To explore the influencing factors for HRQL of welders, multivariate stepwise regression was applied using eight SF-36 dimensions as the dependent variables, and the risk factors in Table 5 as independent variables. The statistical inclusion level of the independent variables was set at 0.05, and the exclusion level was set at 0.10 in the stepwise process. In the Table 6, multivariate stepwise regression results show that native place, monthly income, quantity of children, drinking, sleep time, welding years, welding type, use of personal protective equipment(PPE), great events in life, and some symptoms including dizziness, discomfort of cervical vertebra, low back pain, cough, insomnia were found influential at least one SF-36 dimension of welders.

**Table 5:** Influencing factors and variable coding.

Factors	variable coding
Age	Actual value
Working years	Actual value
Education	Primary school = 1, secondary school = 2, high school = 3
Native place	nonlocal = 1, local = 2
Personal monthly income	¥1000∼¥2000 = 1, ¥2000∼¥3000 = 2, ¥3000∼¥4000 = 3, >¥4000 = 4
Quantity of children	No child = 1, one child = 2, two children = 3, three children or more = 4
smoking	No = 1, Yes = 2
drinking	No = 1, Yes = 2
Family great events	No = 1, Yes = 2
Exercise frequency per week	never = 1, once or twice per week = 2, three times or more per week = 3
Sleep time per night	<7 h = 1, 7 h∼8 h = 2, ≥9 h = 3
Welding type	Non CO_2_ shielded arc welding = 1, CO_2_ shielded arc welding = 2
local ventilation in workplace	No = 1, Yes = 2
Use of personal protective equipment(PPE)	No = 1, Gauze mask = 2, Dust mask = 3
Cough	No = 1, Occasionally = 2, Often = 3
Headache	No = 1, Occasionally = 2, Often = 3
Dizziness	No = 1, Occasionally = 2, Often = 3
Insomnia	No = 1, Occasionally = 2, Often = 3
Discomfort of cervical vertebra	No = 1, Occasionally = 2, Often = 3
Low back pain	No = 1, Occasionally = 2, Often = 3

**Table 6:** Multivariate stepwise regression results of welding group.

Dimension	Factors	Regression coefficient	Standardized regression coefficient	*t*	*P*
PF	Welding years	−0.545	−0.322	−5.812	0.000
	Drinking	−3.594	−0.125	−2.454	0.015
	PPE	3.838	0.120	2.361	0.019
	Dizziness	−2.456	−0.111	−1.824	0.069
	Discomfort of cervical vertebra	−2.500	−0.130	−2.100	0.037
RP	Welding type	−8.379	−0.111	−2.072	0.039
	Native place	−14.507	−0.180	−3.166	0.002
	Low back pain	−8.871	−0.183	−2.622	0.009
	Discomfort of cervical vertebra	−8.524	−0.151	−2.174	0.030
BP	Low back pain	−11.290	−0.428	−6.864	0.000
	Cough	−4.216	−0.136	−2.544	0.011
	Discomfort of cervical vertebra	−4.363	−0.154	−2.562	0.011
GH	Welding years	−0.155	−0.130	−2.211	0.028
	Monthly income	−2.245	−0.233	−4.162	0.000
	Drinking	−2.594	−0.128	−2.250	0.025
	Cough	−2.207	−0.148	−2.496	0.013
VT	Sleep time	7.078	0.174	3.380	0.001
	Low back pain	−7.054	−0.264	−4.429	0.000
	Insomnia	−4.474	−0.140	−2.325	0.021
	Dizziness	−6.138	−0.188	−3.060	0.002
SF	Low back pain	−6.713	−0.267	−4.165	0.000
	Insomnia	−4.328	−0.143	−2.364	0.019
	Cough	−4.178	−0.140	−2.298	0.022
RE	Welding type	−12.450	−0.175	−3.301	0.001
	Quantity of children	5.424	0.134	2.544	0.011
	Native place	−11.967	−0.157	−2.859	0.005
	Insomnia	−10.302	−0.186	−3.226	0.001
	Cough	−10.476	−0.194	−3.350	0.001
MH	Monthly income	−2.228	−0.119	−2.235	0.026
	Sleep time	5.806	0.155	2.900	0.004
	Great events	−4.767	−0.132	−2.426	0.016
	Insomnia	−6.663	−0.225	−3.729	0.000
	Discomfort of cervical vertebra	−4.731	−0.180	−3.068	0.002

Welding years may affect PF and GH, and increasing welding years reduced quality of life in the two dimensions. Welding type may influence RP and RE dimension, CO_2_shieldedarc welding may reduce welders' quality of life in the two dimensions. Some physical symptoms were evidently common risk factors reducing the scores of most of the dimensions, for example, low back pain decreased the dimensions scores in RP, BP, VT and SF; cough reduced the scores in BP, GH, SF and RE dimensions; insomnia affected the scores in dimensions of VT, SF, RE and MH. Among these factors, only the use of PPE, longer sleep time and more children were positive. Longer sleep time per night may increase scores in dimensions of VT and MH; using PPE properly may improve the quality of life in PF dimension; more children resulted in a higher scores in dimensions of RE. In this study, higher personal monthly income was negative in the dimensions of GH and MH. Among these factors, the influence of welding years and low back pain were relatively strong because their standardized regression coefficient were higher than other factors.

## Discussion

The SF-36 is one of the most widely used HRQL forms [18]. It has been referred to as a generic measure since it assesses health concepts that are pertinent to everyone's functional status and well-being, and it can be used in diseased groups as well as general population [19]. Previous studies showed that the SF-36 has a good reliability and validity, which is available for the evaluation of quality of life among Shanghai population [11], [16]. To our best knowledge, study on HRQL among welders is very limited. However, welders expose to multiple occupational hazards in workplaces, and their health status and quality of life are worthy of discussion.

Our results revealed that the welder group had lower scores of SF-36 than the control group in six dimensions except for PF and RE. Meanwhile, CO_2_ arc welders' average scores were significantly lower than that of control group in seven dimensions except for PF, and the scores of RP, GH and RE of CO_2_ arc welder group were also significantly lower than non-CO_2_ arc welder group. The results suggested that welders had worse HRQL, and CO_2_ arc welders' HRQL were affected more deeply. The occupational hazards in CO_2_ arc welding are more serious than most of other type of welding. We had monitored welding fume and manganese and its inorganic compounds by personal sampling method, the results showed that the mean concentration of welding fume and manganese of CO_2_ arc shielded welding was 5.22 mg/m^3^ and 0.221 mg/m^3^ respectively, which was 2.8 times and 2.7 times of non- CO_2_ arc welding group [20]. The type of welding is important with regard to determining the fume composition and generation rate, which together with ventilation patterns will govern the exposure [6].

The HRQL may be influenced by multiple factors. To explore the factors, multivariate stepwise regression was used to identify significant factors related to the eight dimension scores. The results showed that some physical symptoms like discomfort of cervical vertebra, low back pain, cough, and insomnia affected almost all the dimensions of HRQL in welding group. During welding work, welders have to keep an unbalanced position like stoop and squat, and closely contact with welding fume, harmful gas and ultraviolet rays. These occupational hazards may cause impairment of respiratory system, nervous system and musculoskeletal system. All these impairments may cause physical discomforts and affect HRQL of welders.

In addition, welding years and the use of PPE are also influencing factors to HRQL of welders. Welders with longer welding years tend to have lower scores in PF and GH. The use of PPE was related to the dimension of PF, and using dust mask during work is helpful for protecting health and improving quality of life.

Among socio-demographic factors, native place, quantity of children, monthly income were associated with quality of life, especially in dimensions of RP, GH, RE and MH. Quality of life reflects gap between expectation and reality, and the bigger the gap, the worse the quality of life. The results showed that the Shanghai local welders had lower scores in RP and RE than the non-local welders, maybe because the Shanghai local welders have higher expectation, whereas the non-local welders usually come from rural areas of other provinces of China, unlike Shanghai local welders, they have lower expectation and easier to be satisfied. In this study, welders with higher monthly income tend to have lower dimension scores of GH and MH. It may be due to the fact that welders with higher monthly income usually undertake harder work or operate welding longer hours per day. The heavier labor intensity may lead to higher level of exposure to occupational hazards which do harm to their health and affect the HRQL of the welders. There are 19.7% of welders who have more than one child in their family. The results showed that more children in a family induced higher scores in dimension of RE because more children might arouse stronger sense of responsibility of a father.

In the welder group, drinking rate and smoking rate was 26.2% and 37.5% respectively. The multivariate stepwise regression results showed that drinking was a negative factor for the dimension of PF, but smoking was not a significant factors affecting HRQL. Welders should aloof from alcohol and avoid the synergistic effect induced by alcohol and some occupational hazards. As to the relation of smoking with HRQL of welders, a Greek study showed that in a random sample of 472 blue and white-collar heavy industry workers with 57% were current smokers, smoking was a significant hazard and a strong predictor of quality of life [21]. Because our survey sample size is small, and the smoking rate was lower than the abroad study, we temporarily can't find the impact of smoking on the quality of life, so it's not affirmed that smoking has no effect on quality of life of welders.

The results showed that longer sleep time per night may increase scores in dimensions of VT and MH, however, great events in life had negative correlation with the scores of MH dimension. To improve the HRQL, welders should develop a good daily routine and have a sufficient sleep after work. In addition, enterprises should pay attention to employees, and show solicitude for the workers who suffer from important events in life.

Studies on the quality of life of welders are very limited at present. Because the survey was only carried out in Baoshan district of Shanghai city, and the sample size is small, the representativeness may be insufficient. Welder's health related quality of life and its influencing factors still await further research.

## References

[1] Balkhyour MA, Goknil MK (2010) Total fume and metal concentrations during welding in selected factories in Jeddah, Sandi Arabia. Int. J. Environ Res. Public Health 7: 2978–2987 10.3390/ijerph7072978.PMID:20717553 PMC2922740 20717553

[2] 2. Antonini JM, Santamaria AB, Jenkins NT, Albini E, Lucchini R (2006) Fate of manganese associated with the inhalation of welding fumes: Potential neurological effects. NeuroToxicology. 27: : 304–310. PMID:16219356. 10.1016/j.neuro.2005.09.001 16219356

[3] Laohaudomchok W, Lin X, Herrick RF, Fang SC, Cavallari JM (2011) Neuropsychological effects of low-level manganese exposure in welders. Neurotoxicology 32: 171–179 10.1016/j.neuro.2010.12.014.PMID:21192973 21192973 PMC3049839

[4] Bowler RM, Gocheva V, Harris M, Ngo L, Abdelouahab N (2011) Prospective study on neurotoxic effects in manganese-exposed bridge construction welders. Neurotoxicology 32: 596–605 10.1016/j.neuro.2011.06.004.PMID:21762725 21762725

[5] Chang Y, Kim Y, Woo ST, Song HJ, Kim SH (2009) High signal intensity on magnetic resonance imaging is a better predictor of neurobehavioral performances than blood manganese in asymptomatic welders. Neurotoxicology 30: 555–563 10.1016/j.neuro.2009.04.002.PMID:19376157 19376157

[6] Flynn MR, Susi P (2009) Neurological risks associated with manganese exposure from welding operations – A literature review. Int. J. Hyg. Environ Health (212) 459–469 10.1016/j.ijheh.2008.12.003.PMID:19181573 19181573

[7] Chang Y, Jin SU, Kim Y, Shin KM, Lee HJ (2013) Decreased brain volumes in manganese-exposed welders. Neurotoxicology 37: 182–189 10.1016/j.neuro.2013.05.003.PMID:23685157 23685157

[8] Bowler RM, Gysens S, Diamond E, Nakagawa S, Drezgic M (2006) Manganese exposure: Neuropsychological and neurological symptoms and effects in welders. Neurotoxicology. 27: : 315–326. PMID: 16343629. 10.1016/j.neuro.2005.10.007 16343629

[9] Racette BA, Criswell SR, Lundin JI, Hobson A, Seixas N (2012) Increased risk of parkinsonism associated with welding exposure. Neurotoxicology 33: 1356–1361 10.1016/j.neuro.2012.08.011.PMID:22975422 22975422 PMC3651999

[10] Bowler RM, Koller W, Schulz PE (2006) Parkinsonism due to manganism in a welder: Neurological and neuropsychological sequelae. Neurotoxicology. 27: : 327–332. PMID: 16457889. 10.1016/j.neuro.2005.10.011 16457889

[11] Wang R, Wu C, Zhao Y, Yan X, Ma X (2008) Health related quality of life measured by SF-36: a population-based study in Shanghai, China. BMC Public Health 8: 292–299 10.1186/1471-2458-8-292.PMID:18710578 18710578 PMC2527564

[12] Pang SL, Guan WJ, Zhang YS, Xue L, Xie ZS (2004) Analysis on impact factor of life quality in coal miners exposed to dust. *Chinese* Journal of Industrial Medicine [in China] 17: 255–257.

[13] Yu HM, Ren XW, Chen Q, Zhao JY, Zhu TJ (2008) Quality of life of Coal Dust Workers without Pnermoconiosis in mainland China. J Occup Health. 50: : 505–511. PMID: 18946192. 10.1539/joh.l7167 18946192

[14] Ross JA, Macdiarmid JI, Semple S, Watt SJ, Moir G (2013) Cognitive Symptoms and Welding Fume Exposure. Ann Occup. Hyg 57: 26–33 10.1093/annhyg/mes042.PMID:22767555 22767555

[15] Ministry of Health of the People's republic of China (2002) Diagnostic criteria of pneumoconiosis GBZ70-2002 [in China]. Beijing: Law Press, 1–10.

[16] Jin WZ, Yu HT (2012) A study of the reliability and validity of SF-36 scale on evaluating health of population. Chinese Health Resources [in China] 5: 265–267.

[17] Lim LL, Seubsman SA, Sleigh A (2008) Thai SF-36 health survey: tests of data quality, scaling assumptions, reliability and validity in healthy men and women. Health and quality of life outcomes 6: 52 10.1186/1477-7525-6-52.PMID:18634552 18634552 PMC2515296

[18] Ware JE Jr (2000) SF-36 health survey update. Spine. 25: : 3130–3139. PMID: 11124729. 10.1097/00007632-200012150-00008 11124729

[19] Demiral Y, Ergor G, Unal B, Semin S, Akvardar Y (2006) Normative data and discriminative properties of short form 36 (SF-36) in Turkish urban population. BMC public health. 6: : 247–254. PMID: 17029646. 10.1186/1471-2458-6-247 PMC1615878 17029646

[20] Qin JX, Liu WZ, Weng W, Zheng AG (2012) Investigation on concentrations of welding fume, manganese and its inorganic compounds in welding workplace in Baoshan district of Shanghai. Occup and Health [in China]. 28: : 2211–2212, 2215.

[21] Rachiotis G, Behrakis PK, Vasiliou M, Yfantopoulos J (2006) Quality of life and smoking among industrial workers in Greece. Med Lav. 97: : 44–50. PMID: 17009670. 17009670

